# Editorial: Model organisms in aging research: *Drosophila melanogaster*

**DOI:** 10.3389/fragi.2022.1118299

**Published:** 2023-01-10

**Authors:** David Clancy, Stanislava Chtarbanova, Susan Broughton

**Affiliations:** ^1^ Division of Biomedical and Life Sciences, Lancaster University, Lancaster, United Kingdom; ^2^ Department of Biological Sciences, University of Alabama, Tuscaloosa, AL, United States

**Keywords:** *Drosophila*, aging, immunosenescence, microbiota, *C. elegans*, blue light, Mifepristone (RU486)

Eight Nobel prizes have been awarded for work using *Drosophila*, wholly or partly, including Morgan in 1933 (chromosomes), Muller in 1948 (mutations) and Lewis, Weischaus and Nüsslein-Volhard in 1995 (development) and Hoffmann in 2011 (innate immunity). Other highlights include Seymour Benzer basically inventing the field of behaviour genetics in the 1970’s, the *Drosophila* genome sequence in 2000, and the evolution of lifespan in the lab.

Both the genetic dependence and plasticity of ageing were demonstrated when flies selected for later life fertility showed considerably extended lifespan (Rose and Charlesworth 1981). Similar selection experiments investigated the relative importance of mutation accumulation vs. antagonistic pleiotropy in evolution of ageing and lifespan, as well as tradeoffs between longevity and other fitness and metabolic traits (e.g., Partridge, Arking, Luckinbill).

**FIGURE 1:**
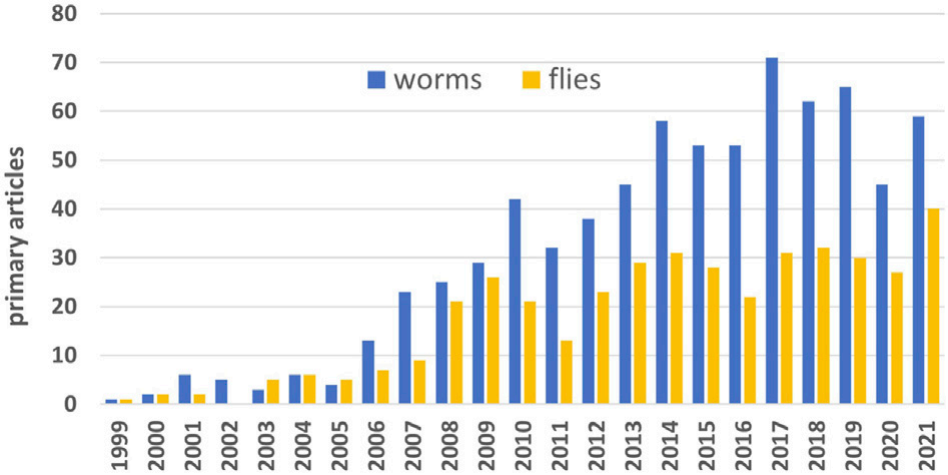
Primary articles by year. Web of Science searched using (“extended lifespan” OR “extending lifespan” OR “extends lifespan” OR “lifespan extension”) AND either *Drosophila* or *elegans* in Research Topic

However over the past two decades *C. elegans* has become the premier invertebrate model organism to study ageing, at least according to publication rates. *C. elegans* overtook *Drosophila* in 2006 and never looked back, in terms of studying lifespan extension (Figure 1), as well as the field of aging more generally. This trend may not be good for the field.

While *Drosophila* has been used partly because of its advantages over mice which are to do with ease of use—relatively short generation time and lifespan, no ethics approvals required, large numbers at low cost—mice also have an advantage over *Drosophila*, shared by *C. elegans*: strains can be stored frozen. To contrast, the *Drosophila* wild type strain Canton-Special (Canton-S), established by C. B. Bridges about 100 years ago, has, at least every month since that time, been transferred to new food manually in countless labs, representing a major cost in labour before we even begin experimentation.

Frozen storage, short lifespan and easy RNAi in *C. elegans* has led to the relative decline of *Drosophila* as a model. Also, *C. elegans* lifespan extensions due to altering gene expression have been large and numerous (623 in *C. elegans* vs. 142 in *D. melanogaster*); so large and so numerous that we might question the relevance to human aging.

*Drosophila* genetics allows knockouts and hypomorphs by mutation, knockdowns by RNAi and overexpression, these last two generally using the GAL4-UAS system or modifications of it. The Genage website shows that 77 of the 142 *Drosophila* genes whose altered expression extended lifespan were accomplished by overexpression. In *C. elegans* the fraction is just 52/623, because mutant strains are numerous, RNAi is easy but overexpression takes more effort. One can accomplish three *C. elegans* lifespan assays in the time it takes to complete one in *Drosophila*. In ageing research, ease of use can be a reason for using a model, but should not the be the primary reason.

Four reports in this Research Topic highlight the range and power of *Drosophila* experimental biogerontology, from microbiota to immunosenescence, to neurodegeneration and experimental lifespan analysis. They illustrate the principle of using a model for what a model should be used for, which is to uncover the fundamental biology of a complex biological process such as aging, that might later be translated in higher organisms.

Mifepristone, a steroidal antiprogestogen, has been used in *Drosophila* to induce gene expression in the Gene-Switch transgenic system since 2001. However, in addition to the therapeutic use of the drug in human birth control and treatment of uterine fibroids, it has more recently been studied for its potential to treat numerous cancers, depression and diabetes. Landis et al. and colleagues present findings in *Drosophila* on the role of Mifepristone and potential interactions with the gut microbiome in fly lifespan. Mifepristone in *Drosophila* extends female lifespan and reduces expression of innate immune response genes. The authors show that lifespan extension in flies due to Mifepristone treatment is not due to an antibiotic effect and suggest that lifespan effects are due to effects of the drug on gene expression. Given the important role of the gut microbiota in human health and the potential therapeutic uses of Mifepristone, Landis et al.‘s paper is a timely and useful contribution to our understanding of the mechanism of action of Mifepristone.

Arias-Rojas et al. and Iatsenko et al. review the role of microbiota in *Drosophila*’s aging. Highlighting the many advantages the *Drosophila* model offers for microbiome research, they provide an overview of the composition and maintenance of the microbiota, its effects on aging and lifespan, and the roles of host genetics and host defenses. They also discuss potential future areas of interest in the field for which *Drosophila* could continue to be model organism of choice: the role of non-bacterial components of the microbiome like fungi and viruses, the role of nutrition, and microbiota-mediated sex-differences in aging.

Corbally and Regan review how *Drosophila* can contribute to the understanding of individual variation in immunosenescence—essential if we are to develop personalized therapeutics to tackle the challenge of ageing human populations with increased susceptibility to pathogens and increased risk of age-related diseases. They discuss the central role played by *Drosophila* in developing evolutionary theories of ageing as well as understanding the genetics of innate immunity, which, along with the functional genetics approaches available in the fly and ease of environmental manipulation, make the fly an ideal model to understand natural variation in immunosenescence.

In their original research article, Yang et al. and colleagues investigated how prolonged exposure to blue light (BL) affects metabolic pathways in *Drosophila* through non-retinal tissue using *eya* ^*2*^ mutants (flies with absent eyes). BL is an environmental factor associated with accelerated aging and impaired mitochondrial function. Exposure changed metabolites associated with energy production. ATP levels were decreased, suggesting that this could lead to accelerated neurodegeneration and death. Indeed, BL exposure decreased lifespan and led to loss of brain tissue, showed deregulation of aspartate and glutamate metabolism and reduced levels of the neurotransmitter gamma-aminobutyric acid (GABA). This study provides novel insights about the impact of BL exposure on evolutionarily conserved metabolic pathways, and the effects on aging and brain integrity.

Long live *Drosophila*.
